# Surgery

**Published:** 1920-03

**Authors:** James Swain


### SURGERY.

The treatment of cases of **traumatic arthritis** by active movements, as advocated by Willems, is a most important substitute for the time-honoured treatment by fixation of the joint, which in pre-war times led to such disastrous results. The writer had an opportunity of seeing many of Dr. Willems's cases in France, and it appeared strange to see patients whose joints were dripping with pus able to move them without difficulty and resulting ultimately in recovery with movable joints such as would not have been possible of attainment under former methods of treatment by immobilisation. A paper[1] by Drs. Clarence A. McWilliams, and William B. Hetzel on a "Report of 82 cases of knee-joint war injuries, with remarks on the Willems treatment by immediate closure and subsequent mobilisation, and the management of the subsequent infection by drainage and mobilisation," contains a clear enunciation of the methods by which Willems has obtained such excellent results.

*General Principles of Willems's Treatment.*—A preliminary X-ray examination of the joint is made to determine the degree of fracture, if any, and to indicate the position of any foreign body. At the operation all damaged tissue surrounding the wound superficial to the synovial membrane is removed. The instruments should then be changed or re-sterilised before the joint is opened up by the removal of the contused edges of the synovial wound. All foreign bodies, portions of clothing or detached fragments of bone should then be removed as quickly as possible, as exposure leads to dryness of the synovial membrane, and this predisposes to ankylosis. This part of the operation should be done without introducing even a gloved finger into the joint. The joint is then washed out with normal saline or other unirritating solution and the edges of the synovial and capsular openings are completely closed with a continuous catgut suture, unless the effusion is distinctly purulent as in neglected cases. The external open wound is loosely packed with gauze wet with Dakin's solution or tubes may be placed in position for treatment by Carrel's method.[2]

No splint should be applied, and any bandage used should not be tight enough to interfere with subsequent movement. At the time the joint is open a culture of its contents is taken for bacteriological examination, and if hæmolytic streptococci are found the joint should at once be drained.

*After-treatment.*—As soon as the patient has recovered from the anæsthetic he is made to *actively* move the joint. If movement is begun thus early the pain is not great, as the periarticular structures have not had time to become infiltrated with inflammatory exudate. The nurse, upon whom much depends, should see that the patient moves the joint as much as he can without causing great pain every two hours night and day. Unless there are displaced bony fragments, when movements are contra-indicated, there is very little pain when active movements are commenced immediately after operation. It is not uncommon for a hemorrhagic effusion to take place into the joint, which should be aspirated in order to prevent any interference with the necessary movements.

In knee-joint cases the patient is made to get out of bed and to take a few steps on the joint without crutches, and almost perfect function is soon restored. It the joint contains pus, made evident either by signs of inflammation or bacteriological examination, thorough drainage must be effected by vertical external or internal incisions, and the joint washed out with Dakin's solution. Tubes are used if drainage appears inefficient, and then the internal ends should only just enter the synovial cavity. The after-treatment is the same as that already given—walking the day after operation without crutches, as the muscular contractions are most useful in causing the expulsion of pus from the joint. The drainage openings are sutured as soon as the discharge becomes serous.

The modifications of the treatment which have been described are as follows: (1) Where there is a bony lesion of middling severity, as in the detachment of an important fragment of an epiphysis. These are the oblique trans-condylar fractures, and there is a danger of the dislocation of the articular line. Immediate mobilisation should be begun, but in the knee walking should not be permitted for about three weeks, in order that sufficient consolidation may take place to prevent displacement of the fracture. (2) Where there are large bony fragments, as in the varieties of T-shaped fractures, the limb should be continuously extended, movements being delayed in order that the articular line may be preserved by consolidation.

The authors desire to note the following summary of the rules which Dr. Willems [3] considers essential in carrying out his method :—

"Active mobilisation should be begun without any delay, and should be carried as far as the patient is in a condition to do it. It should be done without interruption even to the point of fatigue. This mobilisation is not painful in the true sense of the word, it is only laborious, and it is necessary to compel the patient to do it, to tease him if he is lacking in courage.

"It is never necessary to add passive movements to the active movements with the expectation of hastening the process.

"Patients treated by this method never present any serious alteration of their general condition, even during the early febrile period. They never look like those who have been severely infected. They do not fear movements as do those who are immobilised. Even in purulent arthritis it is striking to note that the joint has not the excessive sensibility which an infected joint presents when it is cared for by immobilisation."

**Diverticulitis** is assuming a clinical importance in much the same manner as appendicitis did about thirty years ago. Though known to the few, it has not yet found its way to most of the ordinary text-books, and it has until recently been regarded by the surgeon only from two aspects—as the chief cause of vesico-colic fistula, and as a cause of stenosis of the large bowel resembling malignant disease.

The name is not altogether satisfactory, as diverticula are congenital and acquired, and it is the congenital form with which most people are more familiar, but the acquired form which is affected in the condition under consideration. Dr. W. H. Maxwell Telling, who opened the discussion on this subject at a recent meeting of the Royal Society of Medicine, and whose remarks[4] are taken as the basis of this review, practically restricts the term "diverticulitis" to the "inflammatory changes and secondary pathologic processes generally occurring in or in connection with a certain type of diverticulum. This type is the secondary, acquired, multiple false diverticula of the large bowel, particularly and nearly always found in the sigmoid flexure." It should, however, be borne in mind that acquired diverticula may occur in any part of the alimentary tract. Acquired diverticula are most common after sixty years of age, and it is probable that constipation is an important factor in their production. As Dr. Telling points out, the diverticula are often overlooked because of their smallness, because they mainly enter the fat-laden appendices epiploicæ, and are not discoverable without careful dissection.

*Secondary Processes.*—When once the diverticula have formed the subsequent results depend upon: (1) Being caused by pressure from within they may enlarge; (2) they may harbour fæcal matter or foreign bodies; (3) they tend to undergo secondary pathologic processes. The secondary processes are divided into two groups: A, mainly mechanical, and B, mainly inflammatory, but both forms may co-exist in the same case. A, mechanical: (1) Formation of fæcal concretions in the diverticula; (2) torsion of the diverticulum; (3) lodgment of foreign bodies in the diverticular sac; (4) perforation. B, inflammatory: (1) Diverticulitis (gangrenous, acute, subacute, chronic or latent); (2) passage of organisms without perforation; (3) peridiverticulitis—chronic proliferative inflammation with tendency to stenosis of the bowel; (4) perforation giving rise to general peritonitis, local abscess, fistula or suppuration in a hernial sac; (5) the formation of adhesions, especially to the small intestine, bladder and female pelvic organs; (6) local chronic peritonitis; (7) chronic mesenteritis of the sigmoid loop; (8) metastatic suppuration; (9) the secondary development of carcinoma.

*Symptoms* vary with the pathological condition, but the majority of cases conform to the following types: (1) Inflammatory trouble or tumour in the left lower quadrant of the abdomen; (2) general peritonitis; (3) vesico-colic fistula; (4) pelvic syndromes; (5) intestinal obstruction; (6) mimicry of carcinoma.

About 30 per cent. of cases are associated with tumour or abscess formation, and after thirty years of age the possibility of such swelling being due to diverticulitis should be considered. By far the largest group of cases occurs as a more or less acute inflammatory trouble in the left lower quadrant of the abdomen —analagous to appendicitis on the opposite side.

*Diagnosis.*—The condition which presents the greatest difficulty is the differentiation between carcinoma of the bowel and the peridiverticulitis which exists as a contracting tumour tending towards chronic intestinal obstruction. Even at the time of operation the diagnosis between these conditions may be impossible, but the following points Telling regards as being in favour of peridiverticulitis: (1) The absence of the "shadows of malignancy" from the general condition; (2) tendency to obesity, and maintenance of good nutrition generally; (3) long history of attacks of abdominal pain in the left lower quadrant; (4) history of tumour formation, with subsequent disappearance; (5) absence of blood (visible to naked eye) in stools over a prolonged period; (6) presence of vesical fistula, in which malignancy can be excluded by cystoscopy; (7) negative sigmoidoscopy as regards malignant disease; (8) X-ray demonstration of diverticula; (9) pyrexial attacks; (10) examination of blood showing the presence of neutrophilic leucocytosis, and the absence of the specific nuclear changes characteristic of cancer. Sigmoiditis, hyperplastic tuberculosis, actinomycosis, syphilis and pelvic conditions generally may require consideration in forming a diagnosis.

*Treatment* is by operation unless there is some special contra-indication. All diverticulum-bearing gut should be removed, or recurrence may take place. There seems to be a special liability to post-operative peritonitis in some cases. Care should be taken in handling the gut, lest rupture of a diverticulum should occur. No case of supposed carcinoma of the lower bowel should in future be regarded as inoperable unless diverticulitis has been fully considered.

JAMES SWAIN.

REFERENCES.

[1] *Ann. Surg.*, 1919, lxx., 257.
[2] *Vide Bristol Medico-Chirurgical Journal*, 1917, xxxv. 68.
[3] *Brit. M. J.*, 1920, i. 82; *Lancet*, 1920, i. 85.
[4] *Ann. Surg.*, 1919, lxix. 221.

